# Comparative Study of the Use of HPA Lanolin and Breast Milk for Treating Pain Associated with Nipple Trauma

**DOI:** 10.1055/s-0038-1675180

**Published:** 2018-10-29

**Authors:** Corintio Mariani Neto, Rosemeire Sartori de Albuquerque, Sonia Cristina de Souza, Renata Oliveira Giesta, Andrea Penha Spinola Fernandes, Bárbara Mondin

**Affiliations:** 1 Women’s Health Area, Medicine Course, Universidade Cidade de São Paulo, São Paulo, SP, Brazil; 2 Hospital Maternidade Leonor Mendes de Barros, São Paulo, SP, Brazil; 3 Escola de Artes Ciências e Humanidades, Universidade de São Paulo, São Paulo, SP, Brazil

**Keywords:** nipples/injuries, wound healing/drug effects, lanolin/therapeutic use, breastfeeding/adverse effects, breastfeeding/nipple pain, mamilos/lesões, cicatrização de feridas/efeitos de medicamentos, lanolina/uso terapêutico, aleitamento materno/efeitos adversos, aleitamento/dor no mamilo

## Abstract

**Objective** To compare two different treatments—the use of highly purified anhydrous (HPA) lanolin and expressed breast milk—for women with pain and nipple trauma during the breastfeeding process.

**Method** A total of 180 puerperal women were randomly assigned to 2 groups: one was treated with HPA lanolin and the other with their own expressed breast milk. All of the participants received the same breastfeeding technique instructions and therapeutic care standard. Three assessments were performed: at the time of inclusion in the study (after randomization); after 48 hours; and after 7 days. At each interval, data was collected in relation to pain and trauma. A numerical/verbal category scale was used for the pain variable, and the nipple trauma score for the trauma variable. The results were subjected to statistical analysis using the chi-squared test, the Fisher exact test, the student *t*-test, and the Kolmogorov-Smirnov test. Generalized estimating equations were calculated using the STATA 12 statistical software package (StataCorp LLC, College Station, TX, USA) and IBM SPSS Statistics for Windows, Version 20.0 (IBM Corp, Armonk, NY, USA).

**Results** There was pain improvement from the second to the third assessment in the group that used HPA lanolin, while the pain remained unchanged between these two periods (*p* < 0.001) in the breast milk group. In terms of trauma, improvement was identified in its extension and depth from the first to the third assessment, and it was higher in the HPA lanolin group than in the breast milk group (*p* = 0.025).

**Conclusion** The treatment of pain and nipple trauma with HPA lanolin achieved better results than the one with breast milk, based on a 7-day treatment period.

## Abstract (pt)

**Resumo:**

**Objetivo** Comparar dois tratamentos distintos—o uso de lanolina anidra altamente purificada (HPA, na sigla em inglês) e o próprio leite materno—para mulheres com dor e trauma mamilar durante o processo de amamentação.

**Métodos** Participaram do estudo 180 puérperas randomizadas para 2 grupos: um utilizou tratamento com lanolina HPA e o outro o próprio leite materno. Todas receberam a mesma orientação quanto à técnica de amamentação e rigor terapêutico e foram avaliadas em três momentos: na inclusão no estudo (após a randomização), em 48 horas e em 7 dias. Em todos os momentos, foram colhidos dados em relação à dor e ao trauma. Para a variável dor, utilizou-se escala de categoria numérica/nerbal e, para a variável trauma, a pontuação de traumas mamilares. Os testes estatísticos utilizados foram: Qui-quadrado, teste exato de Fisher, t de Student, e Kolmogorov-Smirnov, sendo que os modelos de equações de estimação generalizadas foram calculados por meio dos pacotes STATA 12 (StataCorp LLC, College Station, TX, EUA) e IBM SPSS Statistics for Windows, Versão 20.0 (IBM Corp, Armonk, NY, EUA).

**Resultados** Houve melhora da dor da segunda para a terceira avaliação no grupo que utilizou a lanolina HPA, enquanto que no grupo que usou leite materno a dor permaneceu inalterada entre esses dois momentos (*p* < 0,001). Quanto ao trauma, foi identificada melhora em sua extensão e profundidade, da primeira para a terceira avaliação, e a melhora foi maior no grupo tratado com lanolina HPA do que no grupo tratado com leite materno (*p* = 0,025).

**Conclusão** O tratamento da dor e do trauma mamilar com lanolina HPA teve melhor resultado comparado com o leite materno, com base em um período de tratamento de 7 dias.

## Introduction

Breastfeeding is the most elementary act for meeting the basic human need for nutrition, but it can be impaired in some situations, especially when women experience nipple trauma or pain when breastfeeding. It has been shown that breast milk is the best nutriment for newborns, and that exclusive breastfeeding for at least 6 months has a direct impact on the social dimension, with improved indicators for maternal and child morbidity and mortality.

An extensive meta-analysis, published in The Lancet, in 2016, by Victora et al,^1^ demonstrated the benefits of breastfeeding for children, including protection against infections and dental malocclusion, higher intelligence, and lower likelihood of being overweight or developing diabetes. For nursing mothers, the benefits include lower incidence of breast cancer, longer intervals between pregnancies, and possible protection against type 2 diabetes and ovarian cancer.^1^

However, for breastfeeding to be effective, certain measures are necessary, primarily related to the adherence to breastfeeding, with instructions for position and latching and treatments in the event of problems. Inadequate breastfeeding can impair the entire process, with effects ranging from changes in the structure of the skin that covers the nipples, causing trauma accompanied by pain (or not), to complications that can make the act of breastfeeding extremely difficult.

In the correct position, the newborn should be close to and facing the mother, with the buttocks supported and the head and body at the same height as the nipple. The lips of the newborn should be facing outward with the mouth semi-open, cheeks rounded and the chin touching the breast. Efficient suction is closely related to a good latch and tissue integrity of the nipple.^2^

Pain with or without trauma may be closely related to an incorrect positioning of the baby in relation to the breast of the mother, indicating the need for a careful assessment by a qualified professional.

A 2013 study by Mariot^3^ involving 342 nursing mothers in a children's hospital in southern Brazil found an 82.5% prevalence of nipple trauma during the breastfeeding process.

In a systematic review by Morland-Schultz et al,^4^ it was shown that the incidence of nipple pain during the breastfeeding stage is of 80% and that, among the various therapies already studied, none has proven to be effective in treatment. Some reduced the pain scores but did not totally resolve the symptom. These studies included lanolin, breast milk, warm moist pads, distilled water, tea bags, hydrogel, gentle massaging only, breastfeeding education only, chlorhexidine alcohol spray, normal care, polyethylene film, and no treatment.

The same authors have also listed studies in which the outcome analyzed was nipple trauma, and they concluded that no single procedure is superior to the others. Studies were reviewed that included no local application, routine care, local heat, vitamin A, anhydrous lanolin, distilled water, tea bags, lanolin USP, hydrogel mixed with breast milk, lanolin cream mixed with breast milk, collagenase, dexpanthenol soap, warm water, nipple protectors, and glycerin gel.^4^

Another important systematic review from the Cochrane Library, by Dennis et al,^5^ examined various substances and procedures for treating nipple pain resulting from trauma and concluded that the evidence was insufficient to determine the best type of treatment for this problem. They have also mentioned an alternative treatment, recently recommended by some professionals, which involves using light emitter diode (LED) phototherapy. Studies of this treatment have suggested that the emission of hyposignals in the nipple facilitates the trauma healing process and accelerates the pain reduction process.

Nipple pain associated with trauma in the breastfeeding process is more common around the 2nd day and usually improves by around the 10^th^ day and it has been noted that, in Canada, it is one of the main reasons for stopping breastfeeding. A study pointed out that 87.3% of the puerperal women started the breastfeeding process and, of these, only 23.1% breastfed their babies up to 6 months, as recommended by the World Health Organization (WHO). A direct relationship between stopping breastfeeding and nipple pain was reported by between 77 and 96% of the women.^5^ ^6^

A study conducted in the United States in 2016 reported that 76.5% of the women started off breastfeeding, but only 49% reached 6 months, and a much smaller proportion (16.4%) breastfed exclusively.^7^

In a Brazilian study, 72.3% of the women breastfed on the 1st day after the birth, dropping to 67% in the first 15 days and to only 9.3% at 180 days. Data from the city of São Paulo was very similar to the national figures: 72% of the women started the breastfeeding process and only 10% continued up to 180 days.^8^

Based on the concerns about breastfeeding-related problems, Martins et al^9^ conducted an experimental study using lanolin to treat surgical wounds in rats. They observed that lanolin in a concentration of up to 2% was not effective in the healing process, mainly in the assessment of outcomes in relation to time. However, in the macroscopic assessment of the group treated with lanolin, the wound had a pinkish border and no dryness, whereas in the control group (normal care), the border was reddish and edematous. The absence of edema in the lanolin group may have been associated with the antiinflammatory action of the eicosapentaenoic and docosahexaenoic acids present in lanolin.

To date, there is insufficient evidence to demonstrate the superiority of a specific substance to prevent or improve nipple trauma and pain during the breastfeeding process. However, La Leche League International, a worldwide organization that provides breastfeeding support, recommends lanolin as the best substance for pure and safe intervention, in that it creates a moist healing environment for nipple trauma and provides a semi-occlusive barrier that promotes retention of internal moisture and prevents dryness. The organization concludes that lanolin can provide a moist environment to prevent skin sores, promote epithelial growth, and decrease nipple pain.^6^

Application of the breast milk of mother on the nipple is another widely used procedure recommended by many professionals and has been adopted as a public policy in various countries. The Canadian Agency for Drugs and Technologies in Health^10^ holds a technical opinion that does not identify any superior substance or procedure to avoid or improve nipple trauma during breastfeeding. It recommends the use of breast milk for prevention and treatment of nipple pain and trauma, after analyzing the conclusions of various randomized and non-randomized studies.

The recommendation of the National Health Service (NHS) in the United Kingdom is based on the most recent Cochrane review of 2014, which did not identify an effective substance for healing nipple trauma and pain during breastfeeding and had no single suggestion for this purpose.^5^

The Australian government published a protocol on nipple trauma during breastfeeding and based its decision on good latching. It argues that correcting the latch will naturally reduce the probability of occurrence of nipple pain and trauma. If a trauma appears, despite having faithfully followed all the steps for breastfeeding, it recommends that mothers apply their own breast milk, which is a natural bacteriostatic lubricant, and let it dry. If pain prevents breastfeeding, it is necessary to remove the milk and administer an oral medication.^11^

Studies come out every year in response to this prevalent problem in the population of puerperal women who have difficulties breastfeeding their babies and, for this reason, are liable to wean early. The present study was performed to contribute to help build knowledge about measures conducive to successful breastfeeding. Its objective was to compare two treatments—the use of highly purified anhydrous (HPA) lanolin versus the mother's own breast milk—for women with nipple pain and trauma during the breastfeeding process.

## Methods

This was a randomized clinical trial that compared two interventions for treating pain associated with nipple trauma during the breastfeeding process. It was conducted at the Hospital Maternidade Leonor Mendes de Barros (HMLMB), a public hospital certified by the Baby-Friendly Hospital Initiative (BFHI) in São Paulo, state of São Paulo, Brazil. Its human milk bank serves, on average, 400 puerperal women per month. Most are from the health service itself, and ∼ 20% are nursing mothers from outside who come to the service due to various breastfeeding problems.

The sample studied was of convenience, due to the number of women attended at the hospital and the period of data collection, from August 2016 to January 2018. It was composed of 180 puerperal women, totaling 90 in the HPA lanolin (Lansinoh Laboratories, Inc., Alexandria, VA, USA) group, called the LA group, and 90 in the breast milk group, called the BM group. Considering a total loss of 15%, a total of 207 puerperal women were included in the study by a simple randomization method (coin flipping): 107 in the BM group and 100 in the LA group with diagnosis of unilateral or bilateral pain and nipple trauma, whose children were healthy, at term, in exclusive breastfeeding. There were 17 losses in the BM group and 10 in the LA group. The main reasons were: no follow-up of the intervention; not allowing the interviewer to enter the house to collect information; or abandonment of exclusive breastfeeding during the study.

To obtain homogeneity between the groups, all the women received guidance on the positioning of the baby and adequate handholding of the nipple-areolar region during breastfeeding. The maternal variables studied were: marital status, self-reported skin color, schooling, primiparity, number of prenatal consultations, gestational age at delivery, type of delivery, presence or absence of skin-to-skin contact in the 1st hour postpartum present, gender of the newborn, nipple type, and type of problem (trauma, pain, or both).

The data collection instrument was specifically designed for the study and previously tested. The data collection started after the study was approved by the Research Ethics Committee of the HMLMB under the number 1.575.758; training of the monitors in the methodological procedures of the study; authorization of the people responsible by the hospital; and signing of the free and informed consent form by the mothers.

The assessments of breastfeeding were scheduled at three points: at enrollment; 48 hours later, and after 7 days. The first assessment was performed at the time of the inclusion of the participants in the study, the second 48 hours later, and the third 7 days later. The instrument was a questionnaire with open-ended and closed questions related to the sociodemographic profile, gestation/puerperium/breastfeeding, and treatment satisfaction of the participants. In relation to pain associated with nipple trauma, an instrument was used to assess intensity and quality of nipple pain through a unidimensional pain assessment scale: a numerical/verbal category scale,^12^ with a range from 0 to 10. For trauma, the nipple trauma score (NTS) (Table 1)^13^ was used, which is a validated instrument for assessing and measuring the evolution of nipple injuries:^13^ ^14^

**Table 1:** Nipple trauma score^13^

Score	
0	No microscopically visible skin changes
1	Erythema or edema or combination of both
2	Superficial damage with or without scab formation on < 25% of the nipple surface
3	Superficial damage with or without scab formation on > 25% of the nipple surface
4	Partial-thickness wound with or without scab formation on < 25% of the nipple surface
5	Partial-thickness wound with or without scab formation on > 25% of the nipple surface

At the time of the first assessment, the diagnosis was performed in relation to pain associated with nipple trauma, manifested by one or more of the following signs: blisters, crusting, erythema, bleeding, swelling, or cracks. This assessment was performed by the team from the human milk bank of the HMLMB and the researchers. After meeting the inclusion criteria and agreeing to participate in the study, a numerical sequence was used, by simple randomization, to determine which group each woman would be assigned to (LA or BM). The instruments were then administered, followed by instructions on the intervention to be adopted.

The BM group received the following instructions: at the end of each feeding, spread a fine layer of breast milk on the region where pain was felt and there was an associated nipple trauma. It was important to wait until it dried naturally before putting on the bra. The LA group was instructed to spread a fine layer of HPA lanolin on the region where pain was felt and there was an associated nipple trauma. It was important to apply a fine layer that covered the whole extension of the trauma and to wait until it dried naturally before putting on the bra. The two groups were also instructed to wash their breasts daily, as well as the bra used, with water and neutral soap; and not to use any type of additional care related to the breast and nipple during the period of the study.

At the time of the second (after 48 hours) and third (after 7 days) assessments of the use of HPA lanolin or breast milk, the women were evaluated through the application of the instruments followed by an orientation.

The data was analyzed descriptively. For the categorical variables, the absolute and relative frequencies were presented, and for the numerical variables, summary measurements (mean, quartile, minimum, maximum and standard deviation [SD]). The existence of associations between two categorical variables was checked using the chi-squared test or, in cases of small samples, the Fisher exact test. The Student *t*-test was used to compare the means of the two treatments, as well as the Kolmogorov-Smirnov test. To analyze the effect of the treatments over time on behavior and on the NTS, generalized estimating equations (GEE)^15^ were used with identity link functions and normal marginal distributions. The approach via GEE, which consists of the generalization of generalized linear models, enables the incorporation of dependency among observations of the same individual resulting from repeated measurements taken over time.

The GEE models were estimated using the STATA 12 statistical software package (StataCorp LLC, College Station, TX, USA). For the other analyses, the software IBM SPSS Statistics for Windows, Version 20.0 (IBM Corp, Armonk, NY, USA) was used.

For all the tests applied, the null hypothesis was that the proportions of the LA group would be equal to those of the BM group; the alternative hypothesis was that the proportions of the LA group would be different from those of the BM group.

## Results

The information and assessments of 180 nursing mothers were analyzed. The mean age was 27.5 years (SD = 6.2 years), ranging from 18 to 48 years old. The mean weight of the newborns was 3,293 g ( ± 512 g). There was no difference between the groups in relation to the age (*p* = 0.648) or newborn weight (*p* = 0.123) variables. The groups were studied by applying statistics tests to assess homogeneity and subsequent comparison (Table 2).

**Table 2:** Distribution of nursing mothers by characteristics, according to treatment

	Total		Treatment				*p-value*
			Lanolin (LA)		Breast milk (BM)		
	*n*	(%)	*n*	(%)	*n*	(%)	
Marital status	180	(100.0)	90	(100.0)	90	(100.0)	0.037
With partner	90	(50.0)	38	(42.2)	52	(57.8)	
Without partner	90	(50.0)	52	(57.8)	38	(42.2)	
Color (self-reported)	180	(100.0)	90	(100.0)	90	(100.0)	0.934^a^
White	93	(51.7)	46	(51.1)	47	(52.2)	
Black/Brown	80	(44.4)	41	(45.6)	39	(43.3)	
Yellow and indigenous	7	(3.9)	3	(3.3)	4	(4.4)	
Schooling	180	(100.0)	90	(100.0)	90	(100.0)	0.900^a^
Elementary education incomplete	8	(4.4)	4	(4.4)	4	(4.4)	
Elementary education complete/secondary education incomplete	29	(16.1)	14	(15.6)	15	(16.7)	
Secondary education complete	93	(51.7)	49	(54.4)	44	(48.9)	
University	50	(27.8)	23	(25.6)	27	(30.0)	
Primiparity	180	(100.0)	90	(100.0)	90	(100.0)	0.881
No	85	(47.2)	42	(46.7)	43	(47.8)	
Yes	95	(52.8)	48	(53.3)	47	(52.2)	
Number of prenatal visits	179	(100.0)	90	(100.0)	89	(100.0)	0.498
≤ 6 visits	25	(14.0)	11	(12.2)	14	(15.7)	
≥ 7 visits	154	(86.0)	79	(87.8)	75	(84.3)	
Gestational age at delivery	171	(100.0)	84	(100.0)	87	(100.0)	0.437
37–38 weeks	22	(12.9)	8	(9.5)	14	(16.1)	
39–40 weeks	123	(71.9)	63	(75.0)	60	(69.0)	
41–42 weeks	26	(15.2)	13	(15.5)	13	(14.9)	
Type of delivery	180	(100.0)	90	(100.0)	90	(100.0)	1.000
Vaginal	92	(51.1)	46	(51.1)	46	(51.1)	
Cesarean section	88	(48.9)	44	(48.9)	44	(48.9)	
Skin-to-skin contact in the delivery room	178	(100.0)	88	(100.0)	90	(100.0)	0.463
Yes	148	(83.1)	75	(85.2)	73	(81.1)	
No	30	(16.9)	13	(14.8)	17	(18.9)	
Gender of the newborn	179	(100.0)	89	(100.0)	90	(100.0)	0.042
Male	80	(44.7)	33	(37.1)	47	(52.2)	
Female	99	(55.3)	56	(62.9)	43	(47.8)	
Type of nipple^b^							
Protruding	97	(96.0)	50	(96.2)	47	(95.9)	1.000^a^
Semi-protruding	76	(98.7)	36	(97.3)	40	(100.0)	0.481^a^
Semi-inverted	6	(66.7)	4	(66.7)	2	(66.7)	1.000^a^
Flat	3	(37.5)	1	(33.3)	2	(40.0)	1.000^a^
Type of problem	180	(100.0)	90	(100.0)	90	(100.0)	1.000^a^
Trauma only	6	(3.3)	3	(3.3)	3	(3.3)	
Pain only	11	(6.1)	5	(5.6)	6	(6.7)	
Trauma and pain	163	(90.6)	82	(91.1)	81	(90.0)	

a *p*-*value* – Descriptive level of the chi-squared test or of the Fisher exact test.

b Multiple response - the total of the percentages does not equal 100%.

Table 2 shows the equivalence among the groups of puerperal women who participated in the study. The prevalences did not differ statistically regarding self-reported skin color, parity, number of prenatal visits, education, type of delivery, gestational age at delivery, and type of nipple. On the other hand, different distributions were identified for marital status and gender of the babies, with a higher percentage of women with partners and of male babies in the BM group. For these two cases, after the evaluation of the adjusted effects, it was noted that there was no interference in the final results. As for skin-to-skin contact and breastfeeding within the first hour after birth, the data showed that there was no significant difference, with a prevalence of breastfeeding with babies placed next to their mothers immediately after birth in both groups.

In both groups, there was a prevalence of protruding nipples and pain associated with trauma in both nipples. The data related to the means and to the confidence interval (CI) of 95% for the pain and trauma (NTS) scores are presented in Table 3.

**Table 3:** Pain and nipple trauma score estimates (mean ± 95% CI) by treatment group. Generalized estimating equations (GEE) model is indicated at the bottom of the table

	Assessment time			*p*-*value*
	Baseline	48 hours	7 days	
Pain				< 0.001
LA	3.8 (3.3–4.2)^A^	5.9 (5.5–6.4)	3.6 (3.2–4.1)^B^	
BM	2.0 (1.6–2.5)^B^	5.4 (5.0–5.9)	5.4 (4.9–5.8)^A^	
NTS				0.460
LA	2.3 (2.1–2.5)	1.9 (1.6–2.1)	0.9 (0.6–1.1)	
BM	2.0 (1.8–2.2)	1.7 (1.5–2.0)	0.8 (0.6–1.1)	

Abbreviations: BM, breast milk group; CI: confidence interval; LA, lanolin group; NTS, nipple trauma score.

*p-value* - Descriptive level for interaction in the GEE model.

(A) and (B) have different means according to contrasts (comparison of groups at each time period).

Effect of time on pain in the breast milk group (*p* < 0.001): baseline < 48 hours = 7 days, and in the lanolin group (*p* < 0.001): baseline = 7 days < 48 hours.

Effect of time on the NTS for the breast milk and lanolin groups (*p* < 0.001): baseline > 48 hour > 7 days.

N = 533 observations in relation to 179 nursing mothers for pain score.

N = 536 observations in relation to 180 nursing mothers for NTS.

Table 3 shows that, over time, different behaviors were noted for the pain score means but not for the NTS between the two groups. In relation to the pain scale, specifically, the mean of the LA group was higher than that of the BM group; both were similar at 48 hours, but at 7 days, the mean of the LA group was lower than that of the BM group. Therefore, in terms of the evolution of pain over time, there was, on average, an increase from the first to the second assessment in both groups. This was not the case at the third assessment, when the BM group maintained the same pain levels, whereas there was a drop in the LA group, with a lower mean pain score than at the first assessment. Table 3 also shows that, in relation to the NTS, both groups had similar means at the three assessments. Regarding the evolution over time, the mean at the first assessment was higher in both groups than at the second assessment, which, in turn, was higher than at the third assessment. Table 4 shows the behavior of the nursing mothers according to the NTS, according to each assessment.

**Table 4:** Distribution of the nursing mothers by nipple trauma score, according to the assessment periods and treatment group

	Lanolin group (LA)						Breast milk group (BM)					
	Baseline		48 hours		7 days		Baseline		48 hours		7 days	
	*n*	(%)	*n*	(%)	*n*	(%)	*n*	(%)	*n*	(%)	*n*	(%)
Score/Total	88	(100.0)	88	(100.0)	88	(100.0)	89	(100.0)	89	(100.0)	89	(100.0)
0 - No microscopically visible skin changes	3	(3.4)	13	(14.8)	45	(51.1)	5	(5.6)	14	(15.7)	49	(55.1)
1 - Erythema or edema or combination of both	16	(18.2)	25	(28.4)	17	(19.3)	19	(21.3)	24	(27.0)	12	(13.5)
2 - Superficial damage with or without scab formation on < 25% of the nipple surface	43	(48.9)	28	(31.8)	22	(25.0)	47	(52.8)	37	(41.6)	23	(25.8)
3 - Superficial damage with or without scab formation on > 25% of the nipple surface	13	(14.8)	13	(14.8)	1	(1.1)	9	(10.1)	7	(7.9)	4	(4.5)
4 - Partial-thickness wound with or without scab formation on < 25% of the nipple surface	5	(5.7)	3	(3.4)	3	(3.4)	6	(6.7)	1	(1.1)	1	(1.1)
5 - Partial-thickness wound with or without scab formation on > 25% of the nipple surface	8	(9.1)	6	(6.8)	0	(0.0)	3	(3.4)	6	(6.7)	0	(0.0)

**Fig. 1:**
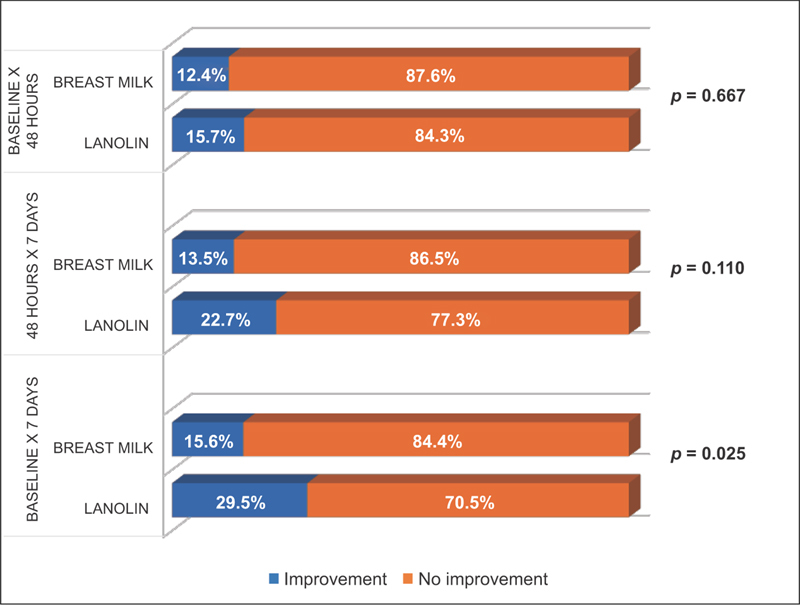
Distribution of improvement by treatment and assessment periods.

To better evaluate the evolution of the severity of trauma through the NTS, in cases of severe trauma with greater extension (score 3—superficial lesion > 25% of the nipple surface, with or without crusting) and depth (scores 4 and 5—partial thickness lesion, with or without crusting), improvement was considered to have occurred if the next scores received were 0 (no visible trauma), 1 (erythema or edema without lesion), or 2 (superficial lesion < 25% of the nipple surface, with or without crusting). Table 5 and Fig. 1 present this distribution by treatment group and assessment periods.

**Table 5:** Distribution of improvement by treatment and assessment periods

	Total		Treatment				*p-*value
			Lanolin group (LA)		Breast milk group (BM)		
	*n*	(%)	*n*	(%)	*n*	(%)	
Baseline × 48 hours	178	(100.0)	89	(100.0)	89	(100.0)	0.667
Improvement	25	(14.0)	14	(15.7)	11	(12.4)	
No improvement	153	(86.0)	75	(84.3)	78	(87.6)	
48 hours × 7 days	177	(100.0)	88	(100.0)	89	(100.0)	0.110
Improvement	32	(18.1)	20	(22.7)	12	(13.5)	
No improvement	145	(81.9)	68	(77.3)	77	(86.5)	
Baseline × 7 days	178	(100.0)	88	(100.0)	90	(100.0)	0.025
Improvement	40	(22.5)	26	(29.5)	14	(15.6)	
No improvement	138	(77.5)	62	(70.5)	76	(84.4)	

*p-*value – descriptive level of the chi-square test.

Table 5 and Fig. 1 illustrate that the improvement percentages from the first assessment period (baseline) to the second (48 hours), and from the second to the third (7 days) were not different in both groups. However, it was noted that the improvement percentage from the first to the third assessment period was almost two times higher in the LA group when compared with the BM group, representing a statistically significant difference.

## Discussion

Studies have shown that there is a higher incidence of nipple pain and injuries in primiparas, fair-skinned women and mothers with male newborns, and that these discomforts appear mainly in the first week after birth.^13^ ^16^ ^17^ ^18^ ^19^ The data in the present study corroborates previous studies in relation to the number of children and skin color, but differs in relation to the gender of the newborn and to the time at which the pain or trauma first occurred, since it was higher for females, and a higher number of nursing mothers reported pain and trauma in both nipples 24 hours after birth.

It is estimated that between 80 and 96% of women experience some degree of pain in the first week after birth. In most cases, the reason is related to a trauma that contributes to giving up breastfeeding.^13^ ^20^ ^21^ ^22^ ^23^

In this sense, studies related to measures to alleviate breastfeeding discomfort have been conducted so that mothers can nurse their babies without having this period marked by suffering due to pain. A review of breastfeeding discomfort showed that there is controversy regarding the prevention of nipple pain and trauma, related to technique and interventions. It has been suggested that a single intervention is not sufficient to improve technique or reduce nipple trauma, but it has also been suggested that further studies be conducted.^24^ Some studies have compared the use of breast milk with the use of HPA lanolin cream for treating nipple trauma and pain in nursing mothers.

In a comparative study, Abou-Dakn et al (2011)^13^ found that there was a significant reduction in breastfeeding-related pain in the group that used HPA lanolin. It was also noted that the trauma healing rates were significantly higher after 14 days of topical treatment, and that benefits appeared after the first 3 days.^13^

In the present study, the results were similar regarding the improvement in pain and trauma, but the results were noted after 7 days of using HPA lanolin. The data regarding pain and trauma were analyzed separately.

In relation to nipple pain in nursing mothers, specifically, the present study identified beneficial effects in the group that used HPA lanolin, with a significant decrease in complaints (*p* < 0.001). The women assigned to the LA group had, on average, more pain than the breast milk group, but after 48 hours the mean pain scores of both groups were similar. Improvement was noted on the 7^th^ day of treatment; there was a considerable reduction in the LA group, with a lower mean pain score than at the baseline of the study, which was not the case with the BM group.

In 2014, another randomized and controlled trial conducted at the University of Toronto, in Canada, assessed the effectiveness of lanolin for treating nipple pain among nursing mothers. Of the 186 participants, 93 were randomized to the treatment group and 93 to normal care. Seven days after the randomization, there was a clinically relevant decrease in nipple pain in both groups. However, there were no statistically significant differences between the groups for other outcomes. Despite these findings, the women in the treatment group were significantly more satisfied with the use of lanolin than those who received normal care.^6^

Pain while breastfeeding has been an object of study for years, and there is a consensus among researchers that its presence discourages women from continuing to breastfeed and can inhibit lactation and significantly undermine the naturalness in the act of breastfeeding.^25^ Apart from the pain, nipple injury, whether associated or not, can aggravate the health condition of the woman and greatly increase the likelihood of early weaning.

A study conducted in 2008 in Brazil among 50 puerperal women with nipple trauma produced similar findings. The use of HPA lanolin yielded statistically significant positive results (*p* < 0.001) in treating women with injuries on both nipples. The size of the injuries decreased at the assessment after 24 hours in the experimental group, compared with the group that used breast milk.^18^

In a controlled randomized trial with 35 nursing mothers in each group, Shanazi et al (2015)^25^ compared the effects of the use of lanolin, peppermint, and dexpanthenol cream for treating nipple trauma in breastfeeding mothers. The results were similar using the three substances, leading to the conclusion that peppermint can be considered an effective option for treating traumatized nipples in patients who want to use plant-based medications.^25^

The present study used the NTS to assess the evolution of the severity of traumas during the period of treatment with breast milk and HPA lanolin. In cases of severe traumas in extension (score = 3) and depth (scores = 4 and 5), improvement was considered to have occurred when the scores dropped to 0, 1, or 2. The results showed that between the first and second assessments, and between the second and third, no difference in improvement between the two groups was noted. However, when the results of the two groups were examined in relation to the improvement between the first and third assessments, after 7 days, the superior results in the group that used HPA lanolin (29.5%), compared with the breast milk group (15.6%) were clear and statistically significant (*p* = 0.025). Therefore, the present study demonstrated that the use of HPA lanolin cream for 7 days improved nipple trauma and promoted the continuation of breastfeeding.

## Conclusion

The present study on the effects of using HPA lanolin compared with breast milk for treating nipple pain and trauma in nursing mothers found a statistically significant improvement in terms of both pain and trauma through the use of HPA lanolin, evidenced more clearly after 7 days of treatment.

## References

[1] Victora C G Bahl R Barros A J Breastfeeding in the 21st century: epidemiology, mechanisms, and lifelong effect Lancet 2016 387(10017):475 490 Doi: 10.1016/S0140-6736(15)01024-7 26869575 10.1016/S0140-6736(15)01024-7

[2] Coca K P Gamba M A de Sousa e Silva R Abrão A CFV Does redingtonite position influence the onset of nipple trauma? Rev Esc Enferm USP 2009 43 02 446 452 Doi:10.1590/S0080-62342009000200026 19655688 10.1590/s0080-62342009000200026

[3] Mariot M DM *Prevalência de Trauma Mamilar em Puérperas de um Hospital Amigo da Criança do Sul do Brasil* [TCC]. Porto Alegre, Brasil: Universidade Federal do Rio Grande do Sul; 2012 http://www.lume.ufrgs.br/bitstream/handle/10183/55306/000857122.pdf?sequence=1. Accessed January 29, 2016.

[4] Morland-Schultz K Hill P D Prevention of and therapies for nipple pain: a systematic review J Obstet Gynecol Neonatal Nurs 2005 34 04 428 437 Doi: 10.1177/0884217505276056 10.1177/0884217505276056 16020410

[5] Dennis C L Jackson K Watson J Interventions for treating painful nipples among breastfeeding women Cochrane Database Syst Rev 2014 12 CD007366 Doi: 10.1002/14651858.CD007366.pub2 25506813 10.1002/14651858.CD007366.pub2 PMC10885851

[6] Jackson K T Dennis C L Lanolin for the treatment of nipple pain in breastfeeding women: a randomized controlled trial Matern Child Nutr 2017 13 03 e12357 Doi: 10.1111/mcn.12357 27477840 10.1111/mcn.12357 PMC6865977

[7] Centers for Disease Control and Prevention. *Breastfeeding Report Card: United States/2013*. Atlanta, GA: CDC; 2013. http://www.cdc.gov/breastfeeding/pdf/2013breastfeedingreportcard.pdf. Accessed February 04, 2016.

[8] Ministério da Saúde. Secretaria de Atenção à Saúde. Departamento de Ações Programáticas e Estratégicas. *II Pesquisa de Prevalência de Aleitamento Materno nas Capitais Brasileiras e Distrito Federal.* Brasília, DF: Ministério da Saúde; 2009 http://www.sbp.com.br/src/uploads/2012/12/pesquisa.pdf. Accessed February 11, 2016.

[9] Martins E F Pereira L M Lima T M Influência da lanolina na cicatrização Saúde Rev. 2005 7 19 25

[10] Canadian Agency for Drugs and Technologies in Health. Management of cracked nipples in breastfeeding women: clinical evidence and guidelines *Rapid Response Report: Summary of Abstracts.* 2010 https://www.cadth.ca/media/pdf/K0292-Nipple-Creams.pdf. Accessed February 12, 2016.

[11] Government of Western Australia. King Edward Memorial Hospital. Women and Newborn Health Service *Clinical Guidelines: Community Midwifery Program: Antenatal Care.* 2015 https://www.kemh.health.wa.gov.au/For-health-professionals/Clinical-guidelines/∼/media/Files/Hospitals/ WNHS/For%20health%20professionals/Clinical%20guidelines/CMP/Antenatal/CMP%20Flowchart%20VBAC%20and%20Risk%20Model.pdf. Accessed February 18, 2016.

[12] Ahmed E MS Mohamed H AEF Abu-Talib Y M Evidence based guideline using to alleviate traumatic nipple among nursing mothers World J Nurs Sci. 2015 1 35 44

[13] Abou-Dakn M Fluhr J W Gensch M Wöckel A Positive effect of HPA lanolin versus expressed breastmilk on painful and damaged nipples during lactation Skin Pharmacol Physiol 2011 24 01 27 35 Doi: 10.1159/000318228 20720454 10.1159/000318228

[14] Vieira F *Efeito da Lanolina Anidra Comparado ao Leite Materno Combinado à Concha de Proteção para Tratamento da Dor e do Trauma Mamilar em Lactantes: Ensaio Clínico Randomizado* [thesis]. Goiânia, Brasil: Universidade Federal de Goiás; 2013 http://pct.capes.gov.br/teses/2013/52001016023P7/TES.PDF. Accessed March 07, 2016.

[15] Zeger S L Liang K Y Longitudinal data analysis for discrete and continuous outcomes Biometrics 1986 42 01 121 130 Doi: 10.2307/2531248 3719049

[16] Mohrbacher N Stock J Nipple problems In: Mohrbacher N Stock J The Breastfeeding Answer Book Schaumburg, IL La Leche League International 1997 387 412

[17] Akkuzu G Taşkin L Impacts of breast-care techniques on prevention of possible postpartum nipple problems Prof Care Mother Child 2000 10 02 38 41 11040764

[18] Coca K P Abrão A CVF Avaliação do efeito da lanolina na cicatrização dos traumas mamilares Acta Paul Enferm 2008 21 11 16 Doi: 10.1590/S0103-21002008000100002

[19] Abrão A CFV Coca K P Pinelli F SG Vieira E Dificuldades no processo de aleitamento materno São Paulo, SP Roca 2009 332 370

[20] Teruya K Bueno L GS Serva V Manejo da lactação São Paulo, SP Atheneu 2009 137 157

[21] Joanna Briggs Institute. The management of nipple pain and/or trauma associated with breastfeeding Aust Nurs J 2009 17 02 32 35 19736722

[22] Coca K P Gamba M A Souza e Silva R Abrão A C Factors associated with nipple trauma in the maternity unit J Pediatr (Rio J) 2009 85 04 341 345 Doi: 10.2223/JPED.1916 19668907 10.2223/JPED.1916

[23] Costa A A Souza E B Guimarães J V Vieira F Evidências das intervenções na prevenção do trauma mamilar na amamentação: revisão integrativa *Rev Eletrônica Enferm.* 2013; 15: 790–801. https://revistas.ufg.br/fen/article/view/22832. Accessed March 07, 2016.

[24] Waldenström U Aarts C Duration of breastfeeding and breastfeeding problems in relation to length of postpartum stay: a longitudinal cohort study of a national Swedish sample Acta Paediatr 2004 93 05 669 676 Doi: 10.1111/j.1651-2227.2004.tb02995.x 15174793

[25] Shanazi M Farshbaf Khalili A Kamalifard M Asghari Jafarabadi M Masoudin K Esmaeli F Comparison of the effects of lanolin, peppermint, and dexpanthenol creams on treatment of traumatic nipples in breastfeeding mothers J Caring Sci 2015 4 04 297 307 Doi: 10.15171/jcs.2015.030 26744729 10.15171/jcs.2015.030 PMC4699508

